# Quercetin protects rats from catheter‐related *Staphylococcus aureus* infections by inhibiting coagulase activity

**DOI:** 10.1111/jcmm.14371

**Published:** 2019-05-15

**Authors:** Lin Wang, BangBang Li, Xiaosa Si, Xingyuan Liu, Xuming Deng, Xiaodi Niu, Yingli Jin, Dacheng Wang, Jianfeng Wang

**Affiliations:** ^1^ Key Laboratory of Zoonosis Research, Ministry of Education College of Veterinary Medicine, Jilin University Changchun Jilin China; ^2^ College of Animal Science Jilin University Changchun Jilin China; ^3^ Department of Pharmacology College of Basic Medical Science, Jilin University Changchun Jilin China; ^4^ The Affiliated Hospital of Qingdao University Qingdao China

**Keywords:** antiinfection, coagulase, quercetin, *Staphylococcus aureus*

## Abstract

Coagulase (Coa) activity is essential for the virulence of *Staphylococcus aureus* (*S aureus*), one of the most important pathogenic bacteria leading to catheter‐related bloodstream infections (CRBSI). We have demonstrated that the mutation of coagulase improved outcomes in disease models of *S aureus* CRBSI, suggesting that targeting Coa may represent a novel antiinfective strategy for CRBSI. Here, we found that quercetin, a natural compound that does not affect *S aureus* viability, could inhibit Coa activity. Chemical biological analysis revealed that the direct engagement of quercetin with the active site (residues Tyr187, Leu221 and His228) of Coa inhibited its activity. Furthermore, treatment with quercetin reduced the retention of bacteria on catheter surfaces, decreased the bacterial load in the kidneys and alleviated kidney abscesses in vivo. These data suggest that antiinfective therapy targeting Coa with quercetin may represent a novel strategy and provide a new leading compound with which to combat bacterial infections.

## 1 INTRODUCTION

Catheter‐related bloodstream infections (CRBSIs) are defined as bloodstream infections often caused by bacteria aggregated on catheters either at the entrance to the bloodstream or inside the bloodstream. Though precautions are taken, there is still a risk of CRBSIs in modern medical institutions.^1^ Bacteria cause CRBSIs in the following three ways: (a) during catheter insertion; (b) during catheter use; (c) or by spreading from a remote infection.^2^ Catheter‐related bloodstream infections is a common, costly and latently lethal complication of catheter use and is a frequent cause of nosocomial bacteremia.^3^ Every year there are approximately 8000 cases of CRBSI that occur in ICUs and 25 000 cases that occur in total; the lethality of CRBSIs can reach 35%.^4^

*Staphylococcus aureus* (*S aureus*) is one of the most important pathogenic bacteria that cause CRBSI; approximately 25%‐30% of CRBSI cases are caused by *S aureus*. When the case occurs in the ICU, the total additional cost of the CRBSI can exceed $30 000.^5^ Infectious complications can appear in patients such as endocarditis, osteomyelitis, abscesses and serious sepsis, which can cause death. During catheter use, in the event of infection with *S aureus*, the probability of developing these complications increases eight‐fold.^6^

In previous studies, methicillin‐resistant *S aureus* (MRSA) was reported to be resistant to vancomycin after the application of methicillin for the treatment of penicillin‐resistant *S aureus*,^7^ emphasizing the essentiality of developing new strategies to control *S aureus* infections. *S aureus* can express a wide array of surface and secreted proteins, one of the most important of which is coagulase (Coa). Coagulase, which is secreted by *S aureus*, has the ability to form clots when inoculated into blood containing heparin.^8^ The cleavage of fibrinogen to fibrin induced by Coa is critical for the establishment of *S aureus* infection.^9^ When a catheter is inserted into a blood vessel, the catheter surface is rapidly coated with fibrinogen. Then, Coa coverts fibrinogen into fibrin fibrils to protect bacteria from opsonophagocytic clearance. This results in pathogens adhering to and remaining on the surface of intravascular catheters, and it is vital to the pathogenicity of CRBSI.^10^

Coagulase is not essential for the growth of *S aureus*; therefore, targeting Coa would reduce the possibility of the development of resistance. In recent years, it has been reported that Coa activity can be inhibited by the thrombin inhibitors dabigatran^11^ and argatroban.^12^ As physiological thrombin inhibitors, they may have side effects, such as the blood not properly coagulating.

In this study, a blood coagulation assay was used to screen for anti‐Coa molecules among approximately 200 natural compounds, and quercetin was found to have effective inhibitory activity against Coa. Quercetin (3,3′,4′,5,7‐pentahydroxyflavone), a five hydroxyl antioxidant flavonoid, processes anti‐inflammatory, anticancer and pro‐metabolic pharmacological functions.^13^ Here, the role of Coa in *S aureus* infection and the potential therapeutic effect of quercetin by inhibiting Coa in CRSBI were further determined.

## 2 MATERIALS AND METHODS

### 2.1 Bacterial strains, plasmids and growth conditions

The bacterial strains and plasmids used in this study are described in Table 1. *Staphylococcus aureus* strains were grown in a brain‐heart infusion medium that was supplemented with chloramphenicol (10 μg/mL) when required. *Escherichia coli* strains were grown in Lysogeny Broth medium that was supplemented with ampicillin (100 μg/mL) when required.

**Table 1:** Strains and plasmids list

Strain or plasmid	Relevant details	Source or reference
Strains		
*S aureus*		
Newman	Wild‐type, srtA positive, haemolysis, coagulase positive	Newman
Δ*coa*	Newman harbors dCas9 and sgRNA‐coa	
*E coli*		
DH5α	supE44 △lacU169 (Φ80 lacZDM15) hsdR17 recA1 endA1 gyrA96 thi‐1 relA1	Invitrogen
BL21	F^−^ *ompT hsdS (rB− mB−) gal dcm* (DE3)	Invitrogen
Plasmids		
pET15b	Expression vector	Amersham
coa‐pET15b	pET15b with *coa* gene	This study

Abbreviations: *S aureus*, *Staphylococcus aureus*; *E coli*, *Escherichia coli*.

### 2.2 Preparation of recombinant Coa

The full‐length coding sequence of mature Coa was cloned into the pET15b vector using the primers Coa_foward_XhoI (GAACTCGAGTCTAGCTTATTTACATGG) and Coa_reverse_BamHI (GTAGGATCCTGGGATAGAGTTACAAAC) to obtain His_6_‐Coa. The Tyr187Ala, Leu221Ala and His228Ala of Coa were generated by site‐directed mutagenesis using the Coa‐pET15b plasmid as a template. The mutagenesis primers were as follows: Tyr187Ala forward: 5′‐AACTAAGGAAGTAGCCGATCTCG‐3′, Tyr187Ala reverse: 5′‐GCTTTATCTTCTTCTGCTGCATTAAAAG‐3′; Leu221Ala forward: 5′‐TTAATCGCTGGAGATACAGACAATC‐3′, Leu221Ala reverse: 5′‐GTCCAGTTTTGCTCGTAACTC‐3′ and His228Ala forward: 5′‐CAATCCAGCTAAAATTACAAATGAACGT‐3′, His228Ala reverse: 5′‐TCTGTATCTCCAAGGATTAAGTC‐3′. All mutations were verified by double‐strand DNA sequencing.

*Escherichia coli* BL21 (DE3) harboring the expression vectors were grown at 37°C and induced with 0.5 mmol/L isopropyl β‐D‐1‐thiogalactopyranoside (IPTG). Following their induction, the cells were centrifuged at 4000 rpm for 30 minutes, suspended in 1× column buffer (0.1 mol/L Tris‐HCl pH 7.5, 0.5 mol/L NaCl) and lysed by an ultrasonic disrupter. The lysates were centrifuged at 12 000 rpm for 1 hour, and the supernatant was subjected to Ni‐NTA affinity chromatography, washed with column buffer with 40 μmol/L imidazole and eluted with 500 μmol/L imidazole. The protein was concentrated and stored at −80°C.

### 2.3 Construction of a Coa deletion mutant of the newman strain

The *coa* gene in the *S aureus* Newman strain was inactivated by allelic exchange as previously described.^14^ Briefly, two DNA fragments were amplified by PCR from the genome of the Newman strain using the primers Down‐srtA‐f (GCGGAATTCCATACAAGAAGCCAAGTAAAAC), Down‐srtA‐r (GCGGGATCCGCTAATGCTAGTAACTTATCTG), Up‐srtA‐f (GCGGTCGACGTATAGCGGATTTTGCAATATAG) and Up‐srtA‐r (GC GCCATGGAATTTTTTAATTCCTCCAAAATG). A 1.5‐kb fragment including the spectinomycin resistance gene was amplified by PCR with the primers Spc‐f (GCGCCATGGGTTCGTGAATACATGTTATA) and Spc‐r (GCGGAATTCGTTTTCTAAAATCTGAT) from the plasmid pSET2s. These three fragments were mixed, digested with EcoRI and NcoI and ligated at 4°C for 1 hour. Using the primers Up‐srtA‐f and Down‐srtA‐r, a 2.0‐kb fragment of the ligation product was amplified by PCR, digested with BamHI and SalI, inserted into pBT2 and used for allele replacement as previously described.^14^ The mutation was confirmed by PCR sequence analysis and Western blotting analysis based on the Newman strain and its Coa mutant. The *coa* knockout strain showed a normal growth rate in Brain Heart Infusion (BHI) broth.

### 2.4 Determination of the minimum inhibitory concentration and growth curves

The minimum inhibitory concentration (MIC) of quercetin against *S aureus* investigated by broth microdilution.^15^ To plot the growth curves of *S aureus*, 1 mL of *S aureus* cultured overnight was added to 50 mL of sterile BHI broth with or without quercetin (256 μg/mL). The absorbance was measured at 600 nm via Infinite^®^ F200 PRO.

### 2.5 Blood coagulation

To evaluate whether quercetin can inhibit the blood coagulation activity of the Coa from *S aureus*, a tube coagulation assay based on fresh rabbit blood containing 1% heparin was performed. Ten microlitres of 100 nmol/L (final concentration) recombinant protein and 10 μL of quercetin at different concentrations were added to 180 μL of blood in small borosilicate glass tubes. Dimethyl Sulfoxide (DMSO) was used as the negative control. The samples were incubated at 37°C, and blood coagulation was verified by inverting the tubes every 10 minutes.

In the plate coagulation assay, 0.9% blood agarose plates were prepared containing 0.4% PEG8000, 3 mg/mL bovine fibrinogen and 1% blood. Small wells were perforated by a gel punch. Twenty microlitres of different concentrations of Coa ranging from 5 to 0.008 mg/mL was added to the wells to determine the activity of Coa. Then, 18 μL of the proper concentration (1 mg/mL) of Coa according to the activity assay was mixed with 2 μL of quercetin at different concentrations (128, 64 and 32 μg/mL, final concentrations) in the holes, and a negative control was performed using 2 μL of DMSO. The coagulation areas were measured after incubation at 37 C for 18 hours.

### 2.6 Thermal shift assay

To verify the interaction between the protein and quercetin, a thermal shift assay was performed. First adequate Cypro‐Orange (5000×) was diluted 100 times and mixed with the target protein on ice. Then, 4 μL of the above sample was mixed with 2 μL of quercetin and 14 μL of protein buffer in PCR tubes. The experiment was measured with the Bio‐Rad iQ5 Real Time PCR system with the following setup: 25°C for 3 minutes (pretreatment); 25‐95°C, raising 1°C per minute; and detection of the fluorescence value. Finally, the curves of the derivative were plotted; the temperature of the derivative curve peak was the Tm value of the protein. Compared with the control protein, a Tm shift >2°C verified the interaction.^16^

### 2.7 Structural modelling of Coa and molecular docking calculation

Using the Autodock4.0 package, the initial coordinates for molecular docking were obtained from the crystal structure of Coa (Protein Data Bank code: 1NU7, 1NU9 and 2A1D).^17^ The molecular geometry optimization in gas phase, vibrational analysis, frontier orbital analysis and Mullikan atomic charges calculation of inhibitors were performed using Gaussian 09 software with the density functional theory method at the B3LYP/6‐311G* level. The initial structures of Coa with its inhibitors for molecular modelling were obtained from molecular docking results. The detailed processes and methods of molecular modelling and calculation of binding free energy are described in our previous literatures.^18^, ^19^

### 2.8 Catheter fibrin deposition in scanning electron microscopy

The previously described catheter fibrin deposition model was used with some minor modifications.^10^ Sterile polyurethane triple lumen central venous catheters (7 French × 16cm, Arrow International) were cut into 2‐mm fragments. These fragments were added to a suspension of either wild‐type (WT) *S aureus* Newman or the *S aureus* Newman Coa mutant at an optical density at 600 nm of 1.0 and incubated on a shaking platform at 37°C for 30 minutes. Then, after being rinsed with 0.9% sterile NaCl, the catheters were placed in 1 mL of fresh rabbit blood spiked with fibrinogen containing heparin and quercetin. After 24 hours, the catheters were rinsed with sterile NaCl 0.9% and fixed overnight. Following a 2‐hour postfixation period in 2% OsO_4_, the samples were sequentially dehydrated in ethanol. After overnight immersion in hexamethyldisilazane, the samples were coated with platina and scanned using a Jeol 7401F scanning electron microscope (Jeol Europe, Zaventem, Belgium) at 2.0 kV.

### 2.9 In vivo catheter infection model

Rats were bred and maintained under specific pathogen‐free conditions. All animal studies were conducted according to the experimental practices and standards approved by the Animal Welfare and Research Ethics Committee at Jilin University. Briefly, female Wistar rats (200‐220 g) were divided into the following three groups: Newman, Newman + quercetin and Δ*coa*, with six rats in each group. These rats were anaesthetized with 10% Nembutal given intraperitoneally. Using cutaneous antisepsis, an incision was made to expose the left external jugular vein. The jugular vein was ligated at the distal end by silk, and a sterile catheter was inserted into jugular vein via a microincision. The catheter was moved into the superiorcaval vein and held the place by proximal and distal ligation with silk stitches. Following the fixation, the catheter was cut and buried subcutaneously. The incisions were closed, and the surgical areas were rinsed by iodophor. Twenty‐four hours after catheter insertion, a suspension of *S aureus* Newman WT or Δ*coa* (5 × 10^9^) was injected into the tail vein. Rats received hypodermic injections of quercetin (100 mg/kg, twice daily) or DMSO 24 hours after bacterial inoculation. Rats were euthanized 7 days after infection, and the catheters were removed aseptically, placed in 1.5 mL tubes with 500 μL of 0.9% sterile NaCl, and sonicated for 10 minutes. The samples were serially diluted and spread on BHI plates. The colony‐forming units (CFUs) were counted after incubation at 37°C overnight. Following cervical dislocation, the right kidneys of the dead rats were excised and weighed. All kidneys were examined for surface lesions. Half of each kidney was homogenized, and serial dilutions were plated as described above for CFU counting. The remaining half of each kidney was sent for histopathology sectioning and haematoxylin‐eosin staining. These slides were evaluated by light microscopy.

### 2.10 Statistical analysis

All experimental data were analysed using GraphPad Prism 5.0 (GraphPad Software). Values are expressed as the mean ± standard error of the mean (SEM, n = 3). The statistical significance was assessed with a one‐way ANOVA (Dunnett's *t* test) and a two‐tailed Student's *t* test. *P* values <0.05 were considered statistically significant.

## 3 RESULTS

### 3.1 Confirmation of the Coa mutant

**Figure 1:**
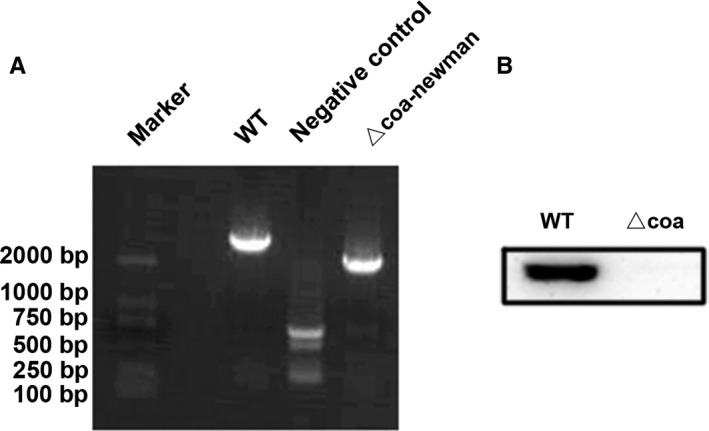
Construction of coagulase (Coa) mutant in the Newman strain. A, PCR products of the parent Newman strain and its mutant stain Δ*coa*. B, Western blotting with anti‐Coa antibody showed the presence of Coa in the wild‐type Newman strain but not in the mutant strain Δ*coa*

To determine the importance of Coa in *S aureus* pathogenesis, homologous recombination was used to generate the Coa mutant in *S aureus* Newman. The Coa mutant was first confirmed by PCR (Figure 1A). Western blotting analysis with an anti‐Coa antibody revealed that the parent Newman stain but not the *coa* mutant expressed Coa (Figure 1B).

### 3.2 Quercetin has no effect on the growth of *S aureus*

**Figure 2:**
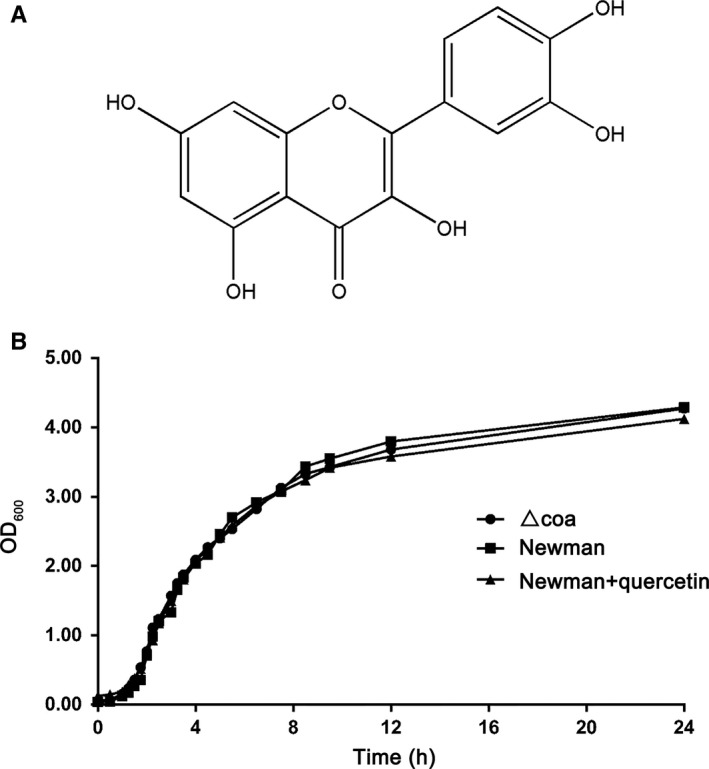
No inhibition of bacterial growth by quercetin. A, Structure of quercetin. B, Growth curves of *Staphylococcus aureus* Newman with or without quercetin (256 μg/mL) and the *S aureus* Newman mutant △*coa*

The MICs of quercetin (Chengdu Herbpurify Co., Ltd., purity > 98%) against both *S aureus* strains (*S aureus* Newman and the Coa mutant) were greater than 1024 µg/mL, suggesting that this compound has no effect on bacterial viability. Furthermore, growth curves of *S aureus* Newman and the Coa mutant showed the same growth state with or without 256 μg/mL quercetin (Figure 2). Taken together, our results revealed that bacterial growth was undisturbed by quercetin even at a concentration of 256 μg/mL.

### 3.3 Quercetin inhibits *S aureus* Coa activity

**Figure 3:**
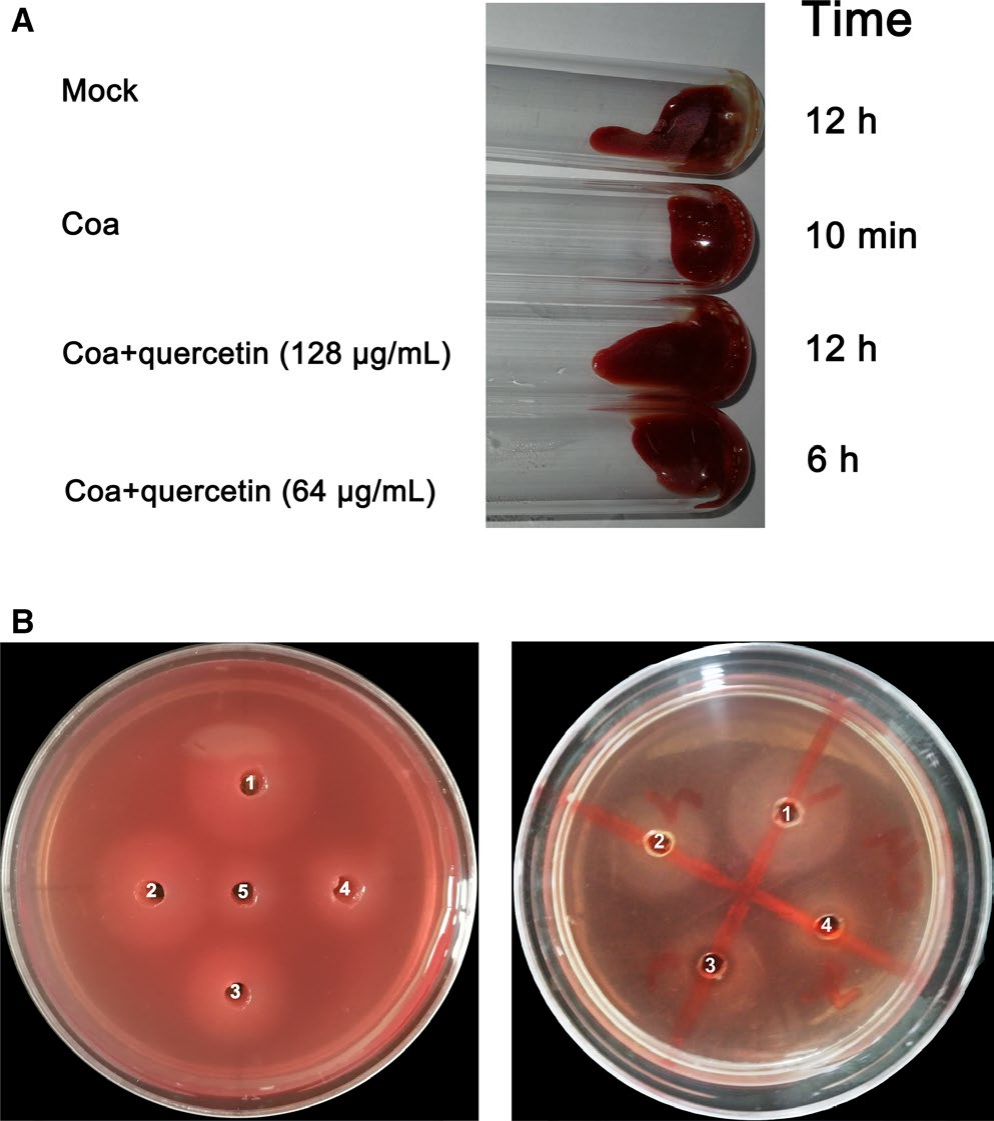
Quercetin inhibits coagulase (Coa) activity. A, Heparin‐treated rabbit blood was mock infected or infected with Coa. Quercetin (64 and 128 μg/mL) was added to the infected blood and incubated at 37°C; the tubes were inverted every 10 min to assess coagulation. B, Left panel: Agarose plate coagulation assay in which 5‐fold dilutions of Coa (well 1, 5 mg/mL; well 2, 1 mg/mL; well 3, 0.2 mg/mL; well 4, 0.04 mg/mL; well 5, 0.008 mg/mL) were added to wells punched in plates containing rabbit blood. Right panel: The appropriate protein concentration (1 mg/mL) was selected to form a suitably sized turbid halo. Coagulase was added to every well, and quercetin (32, 64 and 128 μg/mL, final concentration) was added to wells 2 to 4. The coagulation zones were measured after incubation at 37°C overnight

A test tube coagulation assay was performed to investigate whether quercetin can inhibit Coa activity. Recombinant His_6_‐Coa was purified by affinity chromatography on Ni‐NTA. When Coa (100 nmol/L) was added to rabbit blood with heparin, the blood coagulated within 10 minutes (Figure 3A). However, when recombinant protein was added to rabbit blood mixed with heparin and quercetin, quercetin interfered with the coagulation of the blood. When the concentration of quercetin was 64 μg/mL, the coagulation was prolonged to 6 hours. When the concentration of quercetin was 128 μg/mL, the blood remained without clots for more than 12 hours (Figure 3A).

The plate coagulation assay was further employed to measure the ability of quercetin to inhibit Coa. A turbid halo was seen in the plate due to the change in fibrinogen into a network of fibrin. As shown in the left panel of Figure 3B, the turbid halo increased in size in a concentration‐dependent manner when Coa was added at concentrations ranging from 0.008 to 5 mg/mL. Based on the area of the halo, 1 mg/mL of Coa was chosen for further analysis. Then, each well was supplemented with 1 mg/mL recombinant protein Coa. A serial dilution of quercetin (32, 64 and 128 μg/mL, final concentration) was added to wells 2‐4, and DMSO was added to well 1 as a negative control (Figure 3B, right panel). Consistent with the above results, Coa converted fibrinogen into fibrin. However, the areas of the turbid halos in wells 2‐4 decreased in a concentration‐dependent manner in the presence of quercetin. Taken together, our results established that quercetin can inhibit Coa activity in a concentration‐dependent manner.

### 3.4 The inhibitor quercetin thermally stabilizes Coa

**Figure 4:**
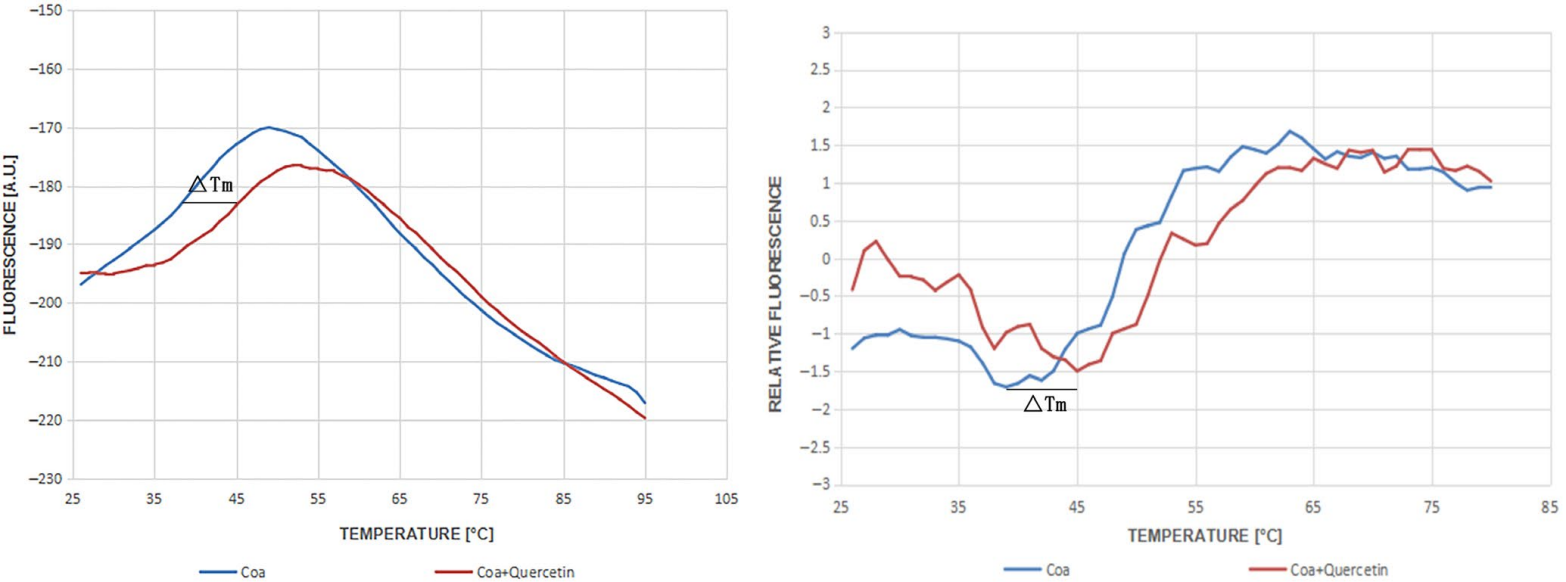
Quercetin thermally stabilizes coagulase (Coa). Coagulase was subjected to a fluorescence‐based thermal shift assay in the presence of 64 μg/mL quercetin (red curves) or buffer only (blue curves) to identify whether quercetin can thermally stabilize Coa

To identify whether quercetin can thermally stabilize Coa, the apparent melting curve was plotted to show the potential binding of fluorescent dye Cypro‐Orange and Coa. The protein starts to unfold when warmed, the dye binds to exposed hydrophobic parts of the protein, and a fluorescent signal is released. The fluorescence intensity reaches a maximum and then starts to decrease. The plotted curve shifts right when the protein becomes more stable. As shown in Figure 4, the blue curve indicates the melting curve of Coa, and the red curve is the melting curve of Coa mixed with quercetin (64 μg/mL). It is notable that the value of Tm changed from 39 to 45°C, representing a significant shift of 6°C (Figure 4). In this study, a shift in ΔTm value greater than 2°C was considered to be significant. Taken together, our results revealed that quercetin could thermally stabilize Coa, suggesting that direct binding occurs between quercetin and Coa.

### 3.5 Determination of the binding mode of Coa with quercetin

**Figure 5:**
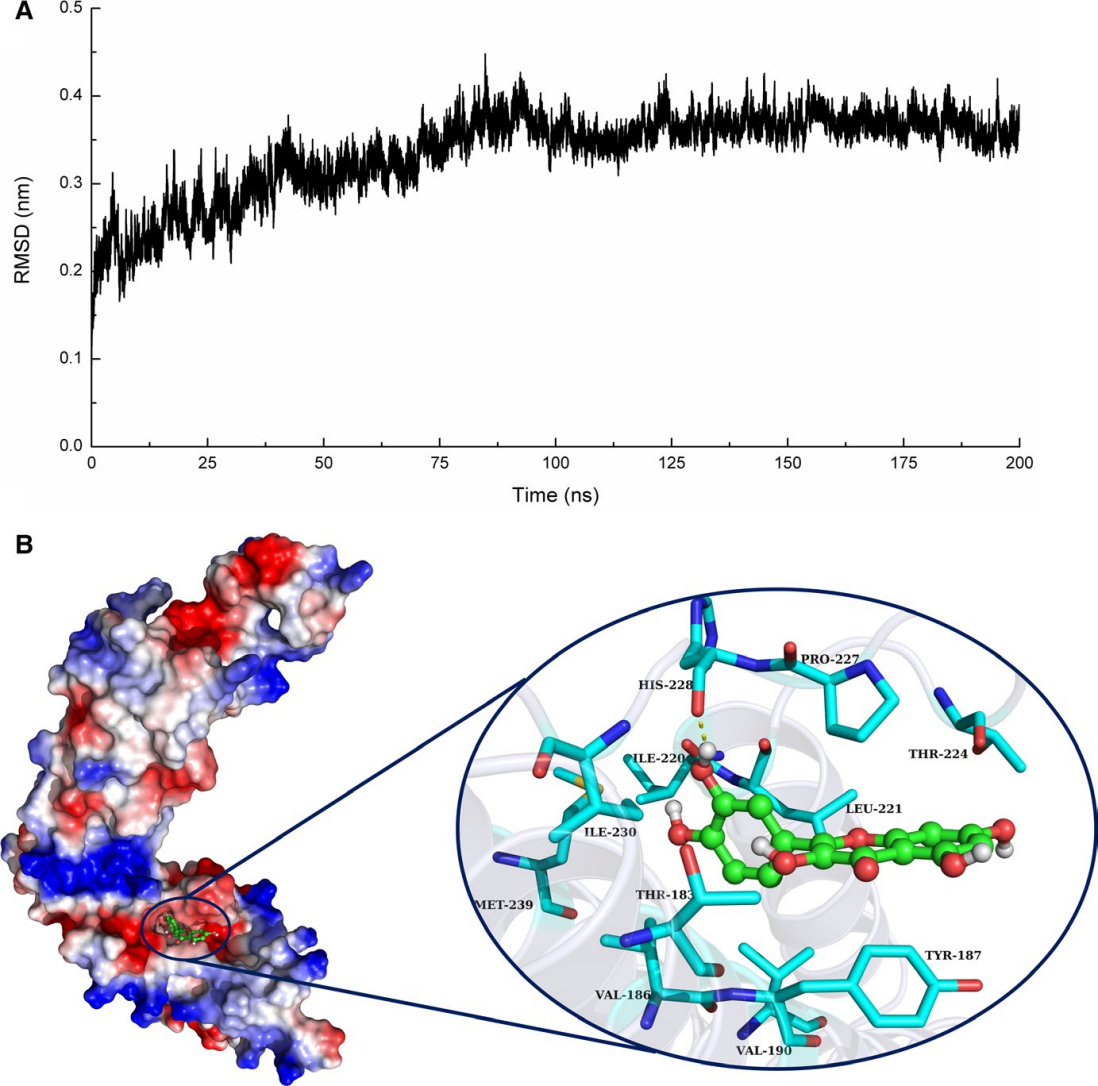
A, The RMSD displayed by the backbone atoms of the protein during MD simulations of coagulase (Coa)‐quercetin is presented. B, The 3D structure determined for the Coa with quercetin complex by the molecular modelling method

Based on the docking results, we performed a 200‐ns molecular dynamics (MD) simulation of Coa‐quercetin complex to determine the preferential binding mode of Coa with quercetin. To explore the dynamic stability of the models and to ensure the rationality of the sampling strategy, the root‐mean‐square deviation (RMSD) values of the protein backbone based on the starting structure throughout the simulation time were calculated and plotted in Figure 5A, indicating that all the protein structures were stabilized during the simulations.

Quercetin acted as a ligand to bind with Coa via intermolecular interactions, and localized to the active site of Coa, over the time course of the simulation. Figure 5B illustrates the predicted binding mode of quercetin to Coa, and the electrostatic potential of the residues surrounding the binding site was plotted using apbs software.^20^ In detail, the binding model of quercetin with the active site of Coa (Figure 5B) revealed that the 4H‐chromen‐4‐onemoietyof quercetin formed strong π‐π interactions with both the benzene ring of Tyr187 and the side chain of Pro227. Moreover, the hydroxyl group of His228 could form a hydrogen bond with quercetin, which was confirmed by energy decomposition analysis (Figure 5B).

The above information indicated that the stabilization of the Coa binding pocket in the complex was mainly due to the residues Tyr187, Pro227 and His228 (Figure 5B).

### 3.6 Identification of the binding site in the Coa‐quercetin complex

**Figure 6:**
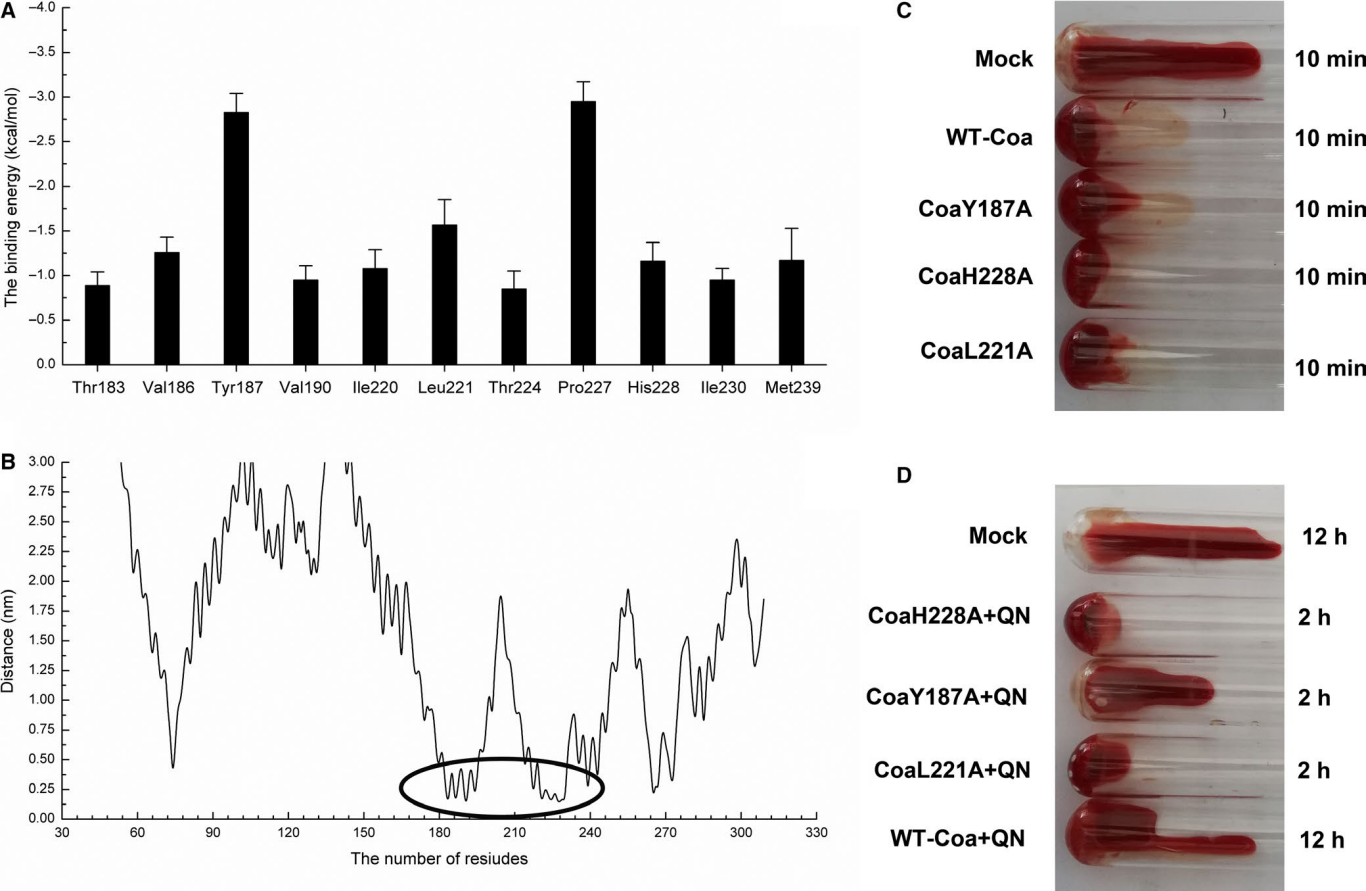
A, Decomposition of the binding energy on a per‐residue basis in the binding sites of the coagulase (Coa)‐quercetin complex. B, The distance between the whole residues of Coa and quercetin. The coagulation activities of WT‐Coa and its mutants in the absence (C) or presence (D) of quercetin

To gain more information about the residues surrounding the binding site and determine their contribution to the whole system, we used the Molecular Mechanics Generalized Born Surface Area (MM‐GBSA) method 21 to calculate the electrostatic, van der Waals, solvation and total contribution of the residues to the binding free energy. The calculation was performed on the 200 MD snapshots taken in the last 10‐ns simulation. The energy contributions from the selected residues are summarized in Figure 6A. In the Coa‐quercetin complex, Tyr187 made an appreciable binding energy contribution, with a Δ*E* of −2.83 kcal/mol (Figure 6A and 6). In fact, Tyr187 was close to the 4H‐chromen‐4‐onemoiety of quercetin, and a strong π‐π interaction existed. Furthermore, residue Pro227 had a Δ*E* of −2.95 kcal/mol, which had strong van der Waals interactions with the ligand because of the close proximity between the residue and quercetin. Moreover, residue Leu221, with a Δ*E* of −1.57 kcal/mol, also strongly interacted with the ligand through van der Waals forces. In addition, His228 had a Δ*E* of −1.16 kcal/mol, due to the formation of a hydrogen bond with quercetin (Figure 6A and B).

The total binding free energies of the Coa‐quercetin complex according to the MM‐GBSA method were summarized in Table 2. For quercetin, the Δ*G*_bind_ was estimated to be −7.93 kcal/mol, indicating that quercetin can bind and interact with the binding site of Coa strongly. To examine the accuracy of the binding site in the Coa‐quercetin complex, the complexes of Y187A‐quercetin, L221A‐quercetin and H228A‐quercetin mutant complexes were used as preliminary structures for MD simulations, and the MD trajectories were continuously analysed with the MM‐GBSA method. The Y187A, L221A and H228A mutants were expressed and purified, and the binding constants between quercetin and the two mutants were investigated by the fluorescence spectroscopy quenching method. The experiment results were highly consistent with those obtained by modelling (WT‐Coa > H28A > Y187A > L221A). Furthermore, the activity of WT Coa and its mutants was almost identical, as evidenced by coagulation was observed for the WT Coa and its mutants (Figure 6C). However, the sensitivity of inhibition of the coagulation of Coa mutants was much lower than the WT Coa (Figure 6D). Thus, consistent with prediction derived from our molecular modelling, quercetin inhibited Coa coagulation by interacting with residues H228, Y187 and L221.

**Table 2:** The binding free energy (kcal/mol) of WT‐LIG, Y187A‐LIG, L221A‐LIG and H228A‐LIG systems based on computational method and the values of the binding constants (*K*_A_) based on the fluorescence spectroscopy quenching

	WT‐Coa	Y187A	L221A	H228A
Computational method	−7.93 ± 1.8	−6.1 ± 1.4	−5.7 ± 1.1	−5.9 ± 1.6
*K*_A_ (1 × 10^4^) L/mol	4.6 ± 1.3	3.2 ± 1.1	3.1 ± 1.2	4.0 ± 1.4

### 3.7 Quercetin reduces adherent biomaterial deposition on catheters in vitro

**Figure 7:**
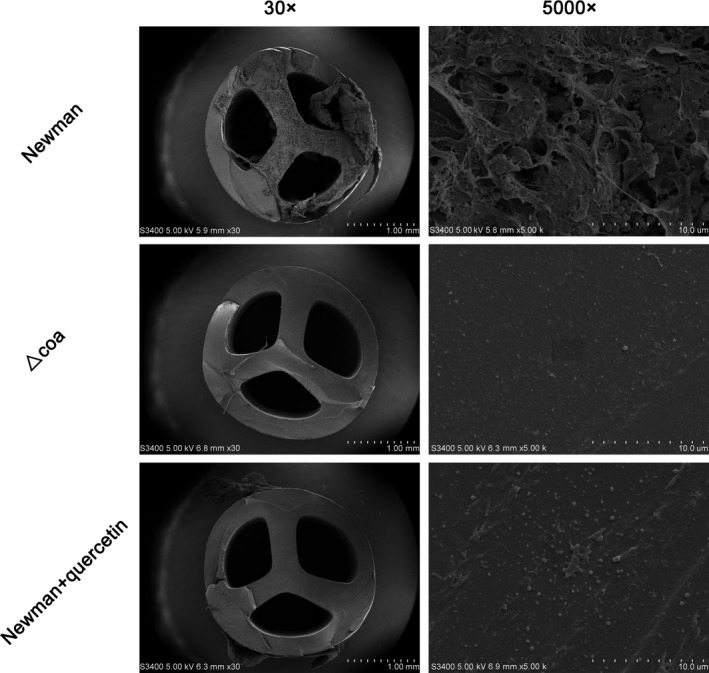
Scanning electron microcopy of *Staphylococcus aureus*‐inoculated catheters. Abundant adherent material was seen on the surfaces of catheters incubated in heparin‐spiked blood for 24 h (top row). Both the absence of coagulase (Coa) (Δ*coa*, middle row) and inhibition with quercetin (*S aureus* treated with quercetin, bottom row) reduced the amount of adherent material, leaving cocci on the surface of the catheter without any surrounding matrix

A catheter fibrin deposition model was used to study whether quercetin could reduce the rapid deposition of adherent biomaterial on the catheter surface. Scanning electron microscopy showed the presence of abundant adherent material on catheter fragments inoculated with *S aureus* Newman WT when they were incubated in heparin‐spiked blood for 24 hours (Figure 7, top row).The adherent biomaterial consisted of fibrin and dispersed cocci.

In the presence of quercetin (256 μg/mL), the adherence of material was significantly reduced, and a small number of cocci were observed to be directly adhered to the catheter surface without any surrounding matrix (Figure 7, bottom row). Similarly, the Δ*coa* mutant was unable to induce rapidly adherent material deposition on the catheter (Figure 7, middle row). Thus, quercetin treatment hinders the deposition of adherent biomaterial on catheters.

### 3.8 Quercetin inhibits catheter colonization and metastatic infectious complications in rats

**Figure 8:**
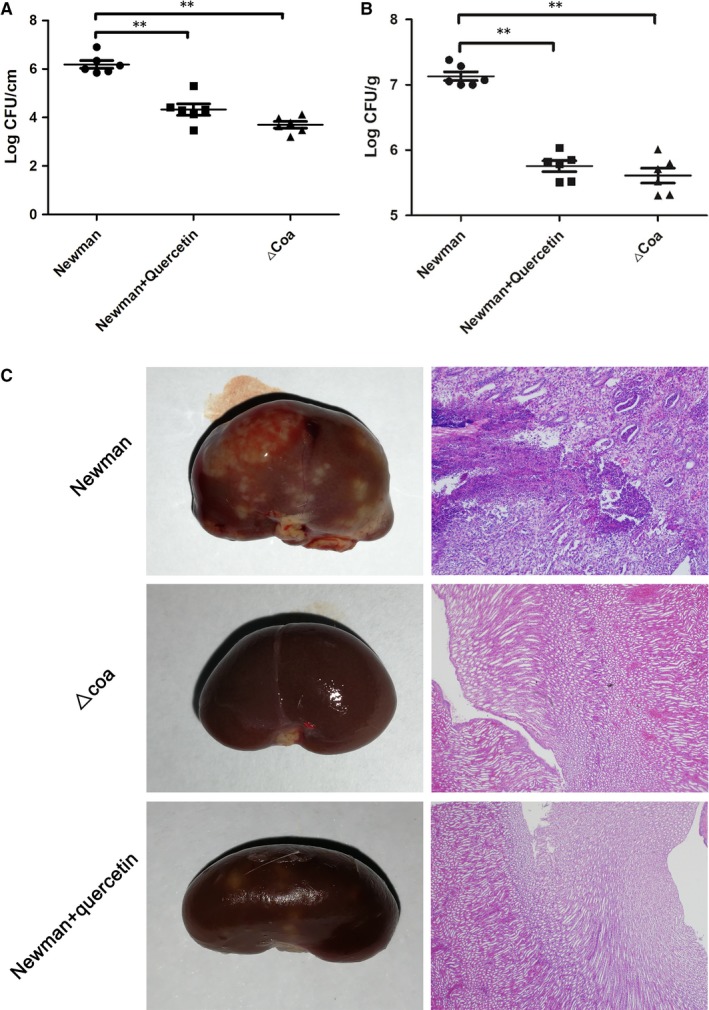
Quercetin protects rats from catheter‐related infections caused by *Staphylococcus aureus*. A rat jugular vein catheter model was employed with infection by 5 × 10^9^ CFUs of *S aureus*. Following treatment with quercetin (100 mg/kg, twice daily) or DMSO for 7 d, the bacterial burdens in the kidneys (B) and on the catheters (C) were determined by the dilution method in Petri dish and the kidneys of euthanized rats were examined by histopathological analysis (C)

To investigate whether quercetin‐mediated protection against catheter‐related infections caused by *S aureus* occurs in vivo, a rat jugular vein catheter model was used. Rats were infected with 5 × 10^9^ CFUs via tail vein injection. Then, rats were hypodermically injected with DMSO or quercetin (twice daily). The Δ*coa* was used as a negative control. The bacterial load was reduced significantly on the catheters in the infected rats treated with quercetin compared with the catheters in those treated with DMSO only (mean load [±SD], 4.33 ± 0.59 log CFU/cm vs 6.19 ± 0.39 log CFU/cm; *P* < 0.01, Figure 8A). Consistent with the bacterial load on the catheters, the bacterial load in the kidneys from the rats treated with quercetin was significantly lower than that in the kidneys from the rats injected with DMSO only (mean load [±SD], 5.76 ± 0.20 log CFU/g vs 7.13 ± 0.16 log CFU/g; *P* < 0.01, Figure 8B). Although a statistically significant reduction was observed for the bacterial load on the catheters between the Newman‐infected rats that received quercetin and the rats that received the mutant Δ*coa* (mean load [±SD], 4.33 ± 0.59 log CFU /cm vs 3.70 ± 0.34 log CFU/cm; *P* < 0.01, Figure 8A), the difference in the bacterial load in the kidneys was not significant (mean load [±SD], 5.76 ± 0.20 log CFU/g vs 5.61 ± 0.28 log CFU/g; *P* > 0.05, Figure 8B).

In agreement with the in vitro results, the kidneys of Newman‐infected rats showed many visible surface abscesses with destruction of the renal unit and obstruction of the renal tubules (Figure 8A). However, significant alleviation of that pathological damage was observed in the infected rats that received quercetin or those infected with the Δ*coa* mutant (Figure 8C). Taken together, our results established that quercetin protected rats from catheter‐related infections by targeting Coa.

## 4 DISCUSSION

Coagulase is characterized as a virulence factor in *S aureus*, one of the important pathogenic bacteria leading to CRBSI.^22^, ^23^ The crystal structure of a fully active Coa fragment (1‐325) bound to prethrombin 2 showed that Coa inserts its Ile‐Val N terminus into the Ile pocket of prethrombin 2, conformationally inducing a functional active site in the cognate zymogen. In addition, the nonproteolytic direct thrombin activation converts fibrinogen into fibrin,^24^, ^25^ suggesting that this virulence factor maybe vital for *S aureus*‐mediated CRBSI.

In this study, we first constructed the *coa* gene knockout strain Δ*coa*, which showed normal bacterial growth rate that was the same as that of the parent strain. However, the Δ*coa* mutant lost the ability to induce adherent material deposition on catheters in vitro. Furthermore, much less bacterial colonization on catheters and pathological injury to kidney occurred in a rat jugular vein catheter model when the rats were infected with the Δ*coa* mutant compared to when the rats were infected with the wild‐type *S aureus*. Therefore, our results suggested that the pathogenicity of the Δ*coa* mutant was significantly decreased in *S aureus* CRBSI. Based on the test tube coagulation assay, we found that the natural compound quercetin was able to inhibit the activity of Coa. Importantly, quercetin at high concentrations did not affect the growth of *S aureus*, indicating that treatment with quercetin would not put selective pressure on *S aureus* and induce bacterial resistance.^26^

Using the apparent melting curve, we further found that quercetin could thermally stabilize Coa with a 6°C shift, suggesting that direct binding may occur between quercetin and Coa. Molecular modelling, site‐specific mutagenesis and fluorescence spectroscopy quenching methods were then used to determine the binding model and inhibitory mechanism of quercetin against Coa. Based on the 200‐ns simulation, quercetin can bind to Coa via intermolecular interactions, and Tyr187, Leu221 and His228 residues contribute critically to the binding of quercetin with Coa. This result was confirmed by site‐specific mutagenesis and fluorescence spectroscopy quenching analysis, suggesting that the experimental data are in good agreement with the results gained from the theoretical calculation. Thus, the residues Tyr187, Leu221 and His228 formed the potential binding site for the engagement of quercetin with Coa.

Consistent with the findings involving the Δ*coa* mutant, treatment with quercetin significantly reduced the fibrin formation on the catheter surfaces induced by *S aureus* in vivo. In addition, the retention of bacteria on catheter surfaces and the bacterial load in the kidneys were significantly decreased following quercetin treatment compared to the control samples without quercetin. In addition, kidney abscesses were alleviated when quercetin was injected intraperitoneally in vivo. In the rat catheter infection model, the bacterial load was reduced by the pharmacological inhibition of Coa by quercetin leading to the decrease in the formation of fibrin depositions on the catheters.

Quercetin, a flavonoid found in plants such as fruits, vegetables and cereals, possesses pharmacological functions including those that are anti‐inflammatory, anticancer and pro‐metabolism.^13^ Here, quercetin was also proven to possess the ability to inhibit *S aureus* virulence by targeting Coa. However, the bioavailability of this flavonoid limits its clinical application due to its relatively low solubility in water.^27^ Thus, the modification of quercetin into increase its solubility is a prerequisite for the clinical application of this flavonoid.

Here, a novel strategy against *S aureus* CRBSI targeting Coa with quercetin was revealed, and this antiinfective therapy would supplement antibiotic therapy without increasing selective pressure on *S aureus* because Coa is not vital factor for bacterial viability.

## CONFLICT OF INTEREST

The authors have no conflict of interest to declare.

## DATA AVAILABILITY STATEMENT

The data used to support the findings of this study are available from the corresponding author upon request.
